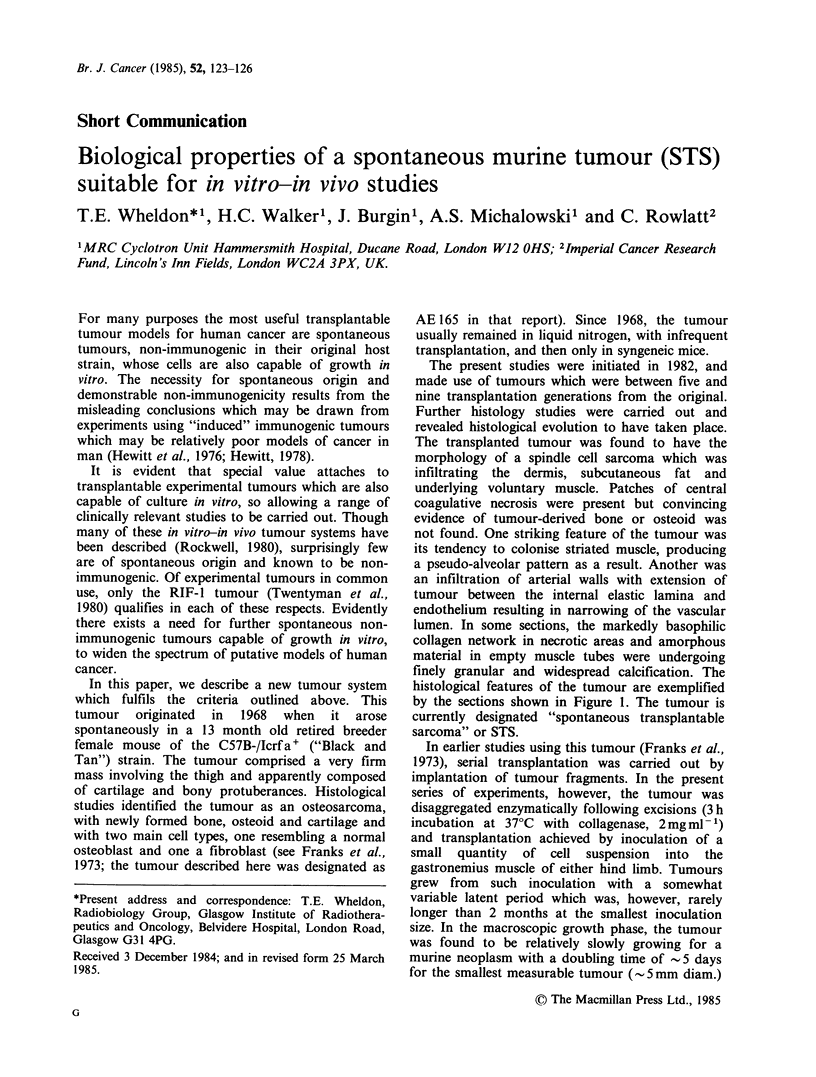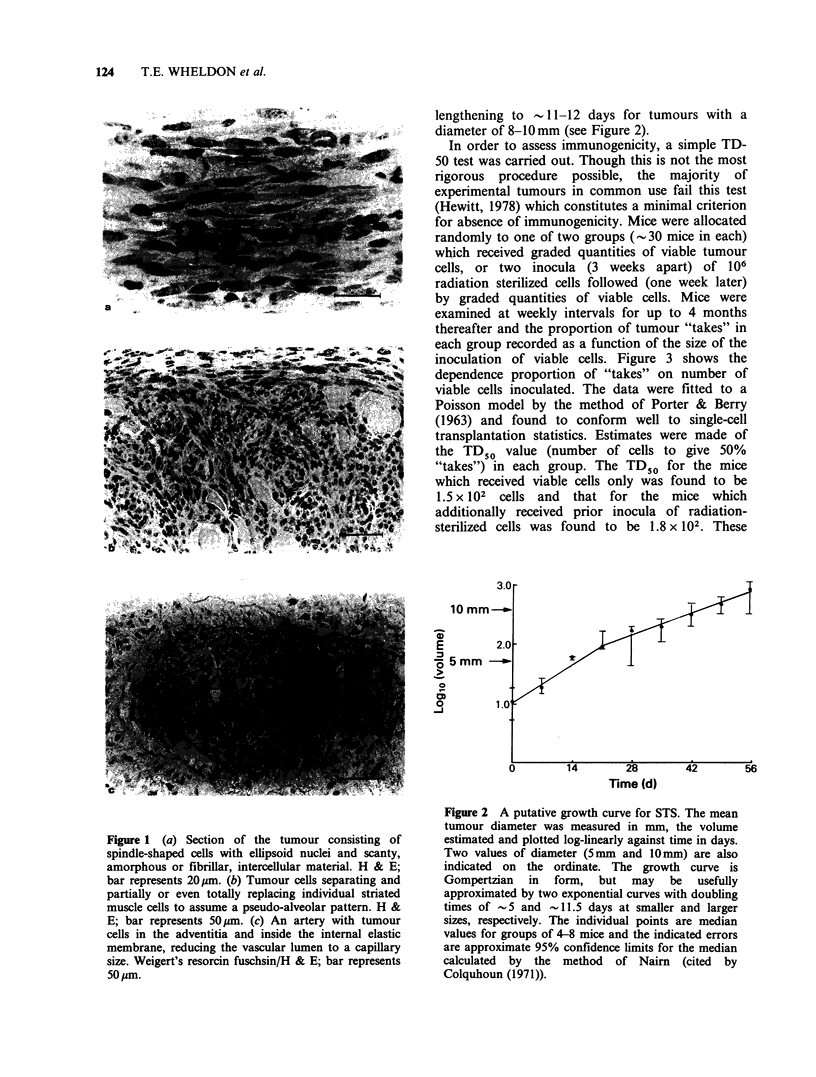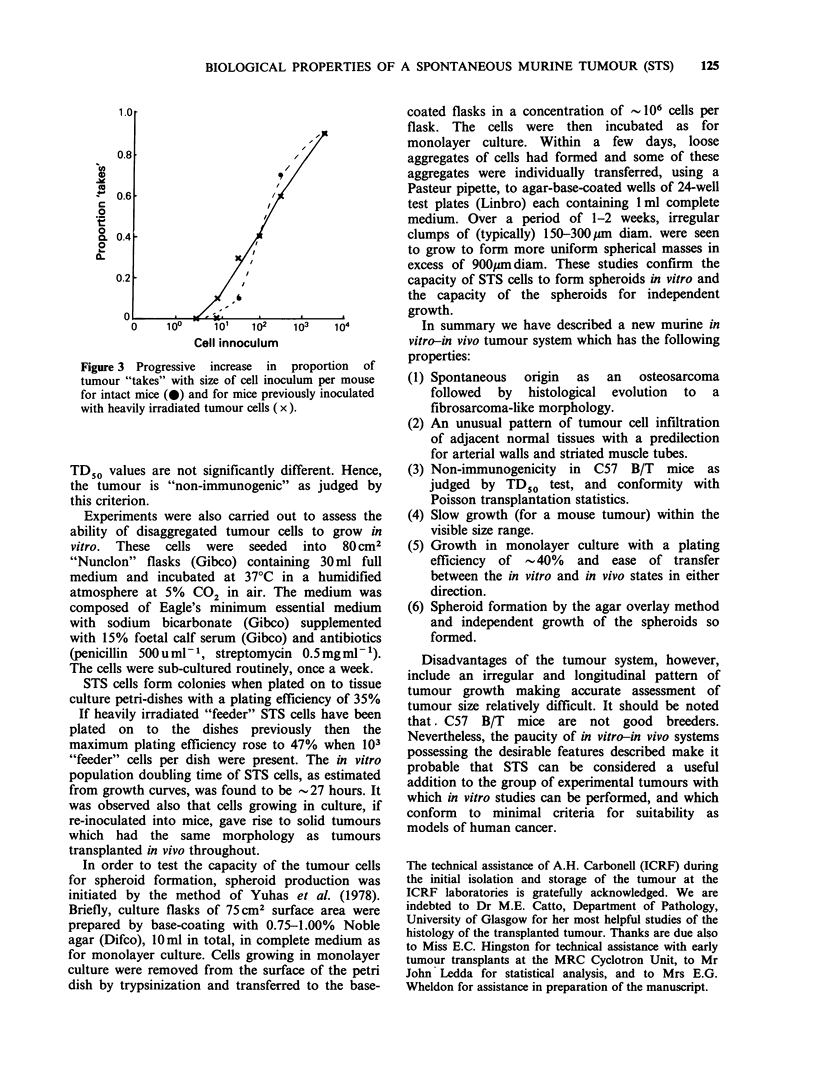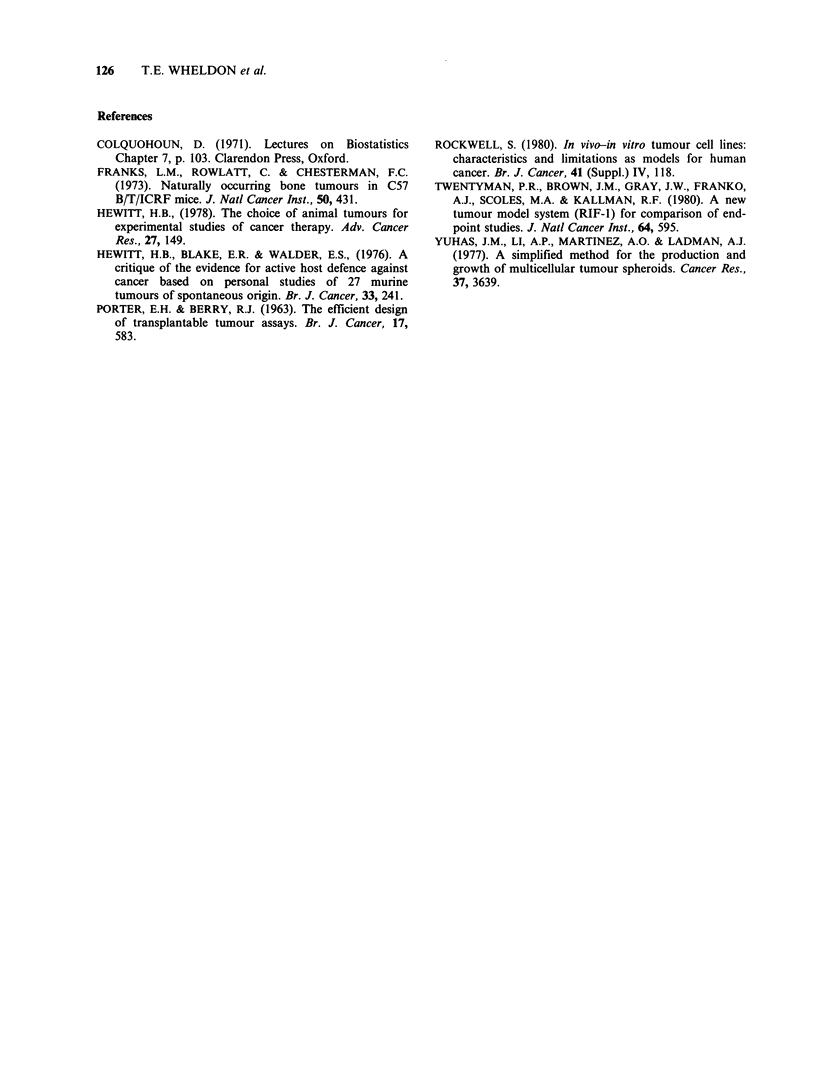# Biological properties of a spontaneous murine tumour (STS) suitable for in vitro-in vivo studies.

**DOI:** 10.1038/bjc.1985.159

**Published:** 1985-07

**Authors:** T. E. Wheldon, H. C. Walker, J. Burgin, A. S. Michalowski, C. Rowlatt

## Abstract

**Images:**


Br. J. Cancer (1985), 52, 123–126

**Short Communication**

### Biological properties of a spontaneous murine tumour (STS) suitable for *in vitro–in vivo* studies

T.E. Wheldon*[1], H.C. Walker[1], J. Burgin[1], A.S. Michalowski[1] and C. Rowlatt[2]

[1]*MRC Cyclotron Unit Hammersmith Hospital, Ducane Road, London W12 0HS;* [2]*Imperial Cancer Research Fund, Lincoln's Inn Fields, London WC2A 3PX, UK.*

For many purposes the most useful transplantable tumour models for human cancer are spontaneous tumours, non-immunogenic in their original host strain, whose cells are also capable of growth *in vitro*. The necessity for spontaneous origin and demonstrable non-immunogenicity results from the misleading conclusions which may be drawn from experiments using "induced" immunogenic tumours which may be relatively poor models of cancer in man (Hewitt *et al.*, 1976; Hewitt, 1978).

It is evident that special value attaches to transplantable experimental tumours which are also capable of culture *in vitro*, so allowing a range of clinically relevant studies to be carried out. Though many of these *in vitro–in vivo* tumour systems have been described (Rockwell, 1980), surprisingly few are of spontaneous origin and known to be non-immunogenic. Of experimental tumours in common use, only the RIF-1 tumour (Twentyman *et al.*, 1980) qualifies in each of these respects. Evidently there exists a need for further spontaneous non-immunogenic tumours capable of growth *in vitro*, to widen the spectrum of putative models of human cancer.

In this paper, we describe a new tumour system which fulfils the criteria outlined above. This tumour originated in 1968 when it arose spontaneously in a 13 month old retired breeder female mouse of the C57B-/Icrf a$^+$ ("Black and Tan") strain. The tumour comprised a very firm mass involving the thigh and apparently composed of cartilage and bony protuberances. Histological studies identified the tumour as an osteosarcoma, with newly formed bone, osteoid and cartilage and with two main cell types, one resembling a normal osteoblast and one a fibroblast (see Franks *et al.*, 1973; the tumour described here was designated as AE165 in that report). Since 1968, the tumour usually remained in liquid nitrogen, with infrequent transplantation, and then only in syngeneic mice.

The present studies were initiated in 1982, and made use of tumours which were between five and nine transplantation generations from the original. Further histology studies were carried out and revealed histological evolution to have taken place. The transplanted tumour was found to have the morphology of a spindle cell sarcoma which was infiltrating the dermis, subcutaneous fat and underlying voluntary muscle. Patches of central coagulative necrosis were present but convincing evidence of tumour-derived bone or osteoid was not found. One striking feature of the tumour was its tendency to colonise striated muscle, producing a pseudo-alveolar pattern as a result. Another was an infiltration of arterial walls with extension of tumour between the internal elastic lamina and endothelium resulting in narrowing of the vascular lumen. In some sections, the markedly basophilic collagen network in necrotic areas and amorphous material in empty muscle tubes were undergoing finely granular and widespread calcification. The histological features of the tumour are exemplified by the sections shown in Figure 1. The tumour is currently designated "spontaneous transplantable sarcoma" or STS.

In earlier studies using this tumour (Franks *et al.*, 1973), serial transplantation was carried out by implantation of tumour fragments. In the present series of experiments, however, the tumour was disaggregated enzymatically following excisions (3 h incubation at 37°C with collagenase, 2 mg ml$^{-1}$) and transplantation achieved by inoculation of a small quantity of cell suspension into the gastronemius muscle of either hind limb. Tumours grew from such inoculation with a somewhat variable latent period which was, however, rarely longer than 2 months at the smallest inoculation size. In the macroscopic growth phase, the tumour was found to be relatively slowly growing for a murine neoplasm with a doubling time of ~5 days for the smallest measurable tumour (~5 mm diam.)

*Present address and correspondence: T.E. Wheldon, Radiobiology Group, Glasgow Institute of Radiotherapeutics and Oncology, Belvidere Hospital, London Road, Glasgow G31 4PG.

Received 3 December 1984; and in revised form 25 March 1985.

© The Macmillan Press Ltd., 1985

Figure 1 (a) Section of the tumour consisting of spindle-shaped cells with ellipsoid nuclei and scanty, amorphous or fibrillar, intercellular material. H & E; bar represents 20 μm. (b) Tumour cells separating and partially or even totally replacing individual striated muscle cells to assume a pseudo-alveolar pattern. H & E; bar represents 50 μm. (c) An artery with tumour cells in the adventitia and inside the internal elastic membrane, reducing the vascular lumen to a capillary size. Weigert's resorcin fuschsin/H & E; bar represents 50 μm.

lengthening to ~11–12 days for tumours with a diameter of 8–10 mm (see Figure 2).

In order to assess immunogenicity, a simple TD-50 test was carried out. Though this is not the most rigorous procedure possible, the majority of experimental tumours in common use fail this test (Hewitt, 1978) which constitutes a minimal criterion for absence of immunogenicity. Mice were allocated randomly to one of two groups (~30 mice in each) which received graded quantities of viable tumour cells, or two inocula (3 weeks apart) of $10^6$ radiation sterilized cells followed (one week later) by graded quantities of viable cells. Mice were examined at weekly intervals for up to 4 months thereafter and the proportion of tumour "takes" in each group recorded as a function of the size of the inoculation of viable cells. Figure 3 shows the dependence proportion of "takes" on number of viable cells inoculated. The data were fitted to a Poisson model by the method of Porter & Berry (1963) and found to conform well to single-cell transplantation statistics. Estimates were made of the $TD_{50}$ value (number of cells to give 50% "takes") in each group. The $TD_{50}$ for the mice which received viable cells only was found to be $1.5 \times 10^2$ cells and that for the mice which additionally received prior inocula of radiation-sterilized cells was found to be $1.8 \times 10^2$. These

Figure 2 A putative growth curve for STS. The mean tumour diameter was measured in mm, the volume estimated and plotted log-linearly against time in days. Two values of diameter (5 mm and 10 mm) are also indicated on the ordinate. The growth curve is Gompertzian in form, but may be usefully approximated by two exponential curves with doubling times of ~5 and ~11.5 days at smaller and larger sizes, respectively. The individual points are median values for groups of 4–8 mice and the indicated errors are approximate 95% confidence limits for the median calculated by the method of Nairn (cited by Colquhoun (1971)).

**Figure 3** Progressive increase in proportion of tumour "takes" with size of cell inoculum per mouse for intact mice (●) and for mice previously inoculated with heavily irradiated tumour cells (×).

$TD_{50}$ values are not significantly different. Hence, the tumour is "non-immunogenic" as judged by this criterion.

Experiments were also carried out to assess the ability of disaggregated tumour cells to grow *in vitro*. These cells were seeded into 80 cm² "Nunclon" flasks (Gibco) containing 30 ml full medium and incubated at 37°C in a humidified atmosphere at 5% $CO_2$ in air. The medium was composed of Eagle's minimum essential medium with sodium bicarbonate (Gibco) supplemented with 15% foetal calf serum (Gibco) and antibiotics (penicillin $500\,u\,ml^{-1}$, streptomycin $0.5\,mg\,ml^{-1}$). The cells were sub-cultured routinely, once a week.

STS cells form colonies when plated on to tissue culture petri-dishes with a plating efficiency of 35% If heavily irradiated "feeder" STS cells have been plated on to the dishes previously then the maximum plating efficiency rose to 47% when $10^3$ "feeder" cells per dish were present. The *in vitro* population doubling time of STS cells, as estimated from growth curves, was found to be ~27 hours. It was observed also that cells growing in culture, if re-inoculated into mice, gave rise to solid tumours which had the same morphology as tumours transplanted *in vivo* throughout.

In order to test the capacity of the tumour cells for spheroid formation, spheroid production was initiated by the method of Yuhas *et al.* (1978). Briefly, culture flasks of 75 cm² surface area were prepared by base-coating with 0.75–1.00% Noble agar (Difco), 10 ml in total, in complete medium as for monolayer culture. Cells growing in monolayer culture were removed from the surface of the petri dish by trypsinization and transferred to the base-coated flasks in a concentration of $\sim 10^6$ cells per flask. The cells were then incubated as for monolayer culture. Within a few days, loose aggregates of cells had formed and some of these aggregates were individually transferred, using a Pasteur pipette, to agar-base-coated wells of 24-well test plates (Linbro) each containing 1 ml complete medium. Over a period of 1–2 weeks, irregular clumps of (typically) 150–300 μm diam. were seen to grow to form more uniform spherical masses in excess of 900μm diam. These studies confirm the capacity of STS cells to form spheroids *in vitro* and the capacity of the spheroids for independent growth.

In summary we have described a new murine *in vitro–in vivo* tumour system which has the following properties:

(1) Spontaneous origin as an osteosarcoma followed by histological evolution to a fibrosarcoma-like morphology.
(2) An unusual pattern of tumour cell infiltration of adjacent normal tissues with a predilection for arterial walls and striated muscle tubes.
(3) Non-immunogenicity in C57 B/T mice as judged by $TD_{50}$ test, and conformity with Poisson transplantation statistics.
(4) Slow growth (for a mouse tumour) within the visible size range.
(5) Growth in monolayer culture with a plating efficiency of ~40% and ease of transfer between the *in vitro* and *in vivo* states in either direction.
(6) Spheroid formation by the agar overlay method and independent growth of the spheroids so formed.

Disadvantages of the tumour system, however, include an irregular and longitudinal pattern of tumour growth making accurate assessment of tumour size relatively difficult. It should be noted that. C57 B/T mice are not good breeders. Nevertheless, the paucity of *in vitro–in vivo* systems possessing the desirable features described make it probable that STS can be considered a useful addition to the group of experimental tumours with which *in vitro* studies can be performed, and which conform to minimal criteria for suitability as models of human cancer.

The technical assistance of A.H. Carbonell (ICRF) during the initial isolation and storage of the tumour at the ICRF laboratories is gratefully acknowledged. We are indebted to Dr M.E. Catto, Department of Pathology, University of Glasgow for her most helpful studies of the histology of the transplanted tumour. Thanks are due also to Miss E.C. Hingston for technical assistance with early tumour transplants at the MRC Cyclotron Unit, to Mr John Ledda for statistical analysis, and to Mrs E.G. Wheldon for assistance in preparation of the manuscript.

### References

COLQUOHOUN, D. (1971). Lectures on Biostatistics Chapter 7, p. 103. Clarendon Press, Oxford.

FRANKS, L.M., ROWLATT, C. & CHESTERMAN, F.C. (1973). Naturally occurring bone tumours in C57 B/T/ICRF mice. *J. Natl Cancer Inst.*, **50**, 431.

HEWITT, H.B., (1978). The choice of animal tumours for experimental studies of cancer therapy. *Adv. Cancer Res.*, **27**, 149.

HEWITT, H.B., BLAKE, E.R. & WALDER, E.S., (1976). A critique of the evidence for active host defence against cancer based on personal studies of 27 murine tumours of spontaneous origin. *Br. J. Cancer*, **33**, 241.

PORTER, E.H. & BERRY, R.J. (1963). The efficient design of transplantable tumour assays. *Br. J. Cancer*, **17**, 583.

ROCKWELL, S. (1980). *In vivo–in vitro* tumour cell lines: characteristics and limitations as models for human cancer. *Br. J. Cancer*, **41** (Suppl.) IV, 118.

TWENTYMAN, P.R., BROWN, J.M., GRAY, J.W., FRANKO, A.J., SCOLES, M.A. & KALLMAN, R.F. (1980). A new tumour model system (RIF-1) for comparison of end-point studies. *J. Natl Cancer Inst.*, **64**, 595.

YUHAS, J.M., LI, A.P., MARTINEZ, A.O. & LADMAN, A.J. (1977). A simplified method for the production and growth of multicellular tumour spheroids. *Cancer Res.*, **37**, 3639.